# Exclusive detection of cerebral hemodynamics in functional near-infrared spectroscopy by reflectance modulation of the scalp surface

**DOI:** 10.1117/1.JBO.25.8.087001

**Published:** 2020-08-05

**Authors:** Hiroshi Kawaguchi, Yukari Tanikawa, Toru Yamada

**Affiliations:** National Institute of Advanced Industrial Science and Technology (AIST), Human Informatics and Interaction Research Institute, Tsukuba, Japan

**Keywords:** functional near-infrared spectroscopy, partial optical path length, reflectance modulation, scalp blood flow, signal contamination

## Abstract

**Significance:** Functional near-infrared spectroscopy (fNIRS) is a technique for detecting regional hemodynamic responses associated with neural activation in the cerebral cortex. The absorption changes due to hemodynamic changes in the scalp cause considerable signal contamination in the fNIRS measurement. A method for extracting hemodynamic changes in the cerebral tissue is required for reliable fNIRS measurement.

**Aim:** To exclusively detect cerebral functional hemodynamic changes, we developed an fNIRS technique using reflectance modulation of the scalp surface.

**Approach:** The theoretical feasibility of the proposed method was proven by a simulation calculation of light propagation. Its practical feasibility was evaluated by a phantom experiment and brain activation simulation mimicking human fNIRS experiments.

**Results:** The simulation calculation revealed that the partial path length of the scalp was changed by reflectance modulation of the scalp surface. The influence of absorption change in the superficial layer was successfully reduced by the proposed method, using only measurement data, in the phantom experiment. The proposed method was applicable to human experiments of standard designs, achieving statistical significance within an acceptable experimental time-frame.

**Conclusions:** Removal of the scalp hemodynamic effect by the proposed technique will increase the quality of fNIRS data, particularly in measurements in neonates and infants that typically would require a dense optode arrangement.

## 1 Introduction

Functional near-infrared spectroscopy (fNIRS) is a relatively simple technique for detecting the regional hemodynamic response associated with neural activation in the cerebral cortex. Compared to other elaborate functional neuroimaging techniques, such as functional magnetic resonance imaging and positron-emission tomography, fNIRS has the advantages of low risks, as it does not involve exposure to a magnetic field or radiation, hardware portability, tolerance for electromagnetic noise, and lower running cost. These advantages of fNIRS have increased its use in various disciplines, but some technical concerns in fNIRS have remained, which have not been disclosed to such users.^1^^,^^2^ These technical concerns include its sparser sampling than the localization of cerebral functions,^3^ the variation in noise with hair coverage,^4^ optode artifacts,^5^^–^^7^ and the influence of scalp blood flow changes.^8^^–^^12^

The influence of scalp blood flow changes on the measured signal is a fairly well-known issue. In fNIRS measurement, pairs of irradiation and detection optodes are fixed to the surface of the subject’s head. The light from the irradiation optode propagates into the head tissues, which are comprised of several tissue layers with different optical properties, such as the scalp, skull, cerebrospinal fluid (CSF), and gray matter and white matter cerebral tissues. The detection optode receives some of the light propagated in these tissues; thus, the detected light inevitably has transited not only the cerebral tissues but also other tissues, including the scalp. If a temporal change in optical properties occurs only in the cerebral tissues, the fNIRS signal might contain only the cerebral hemodynamic response. However, changes in systemic physiological activities, including changes in blood pressure, heart rate, respiration, physical posture, and vasomotion, often induce changes in scalp blood flow. Since the fNIRS measurement sensitivity at the scalp layer is about 10 times larger than that of the gray matter layer,^11^^,^^13^ the optical property changes brought about through scalp blood flow changes cause considerable signal contamination in fNIRS measurement. Therefore, a method for extracting optical property changes in the cerebral tissue layer is required for reliable fNIRS measurement.

Several methods have been proposed to remove the signal contamination originating from the scalp tissue; however, these methods have some drawbacks in terms of practical implementation. Some methods have introduced additional optodes, with a closer interoptode distance, mainly for detecting scalp blood flow changes.^8^^,^^9^^,^^14^^–^^18^ While this has reduced signal contamination in fNIRS measurement to some extent, it has also made it difficult to achieve a sufficiently dense sampling of fNIRS signals commensurate with cerebral functional localization. The consequent sparse spatial sampling in fNIRS measurement may easily result in oversight of cortical activation localized in a gyrus.^3^ Some other methods have employed another strategy that extracts signals based on spatial or temporal prior knowledge for the functional hemodynamic response, such as spatial localization,^19^ informational independence from contaminants,^20^ correlation with the task period,^21^^,^^22^ and correlation between two hemoglobin species.^12^^,^^23^ Since these methods can be applied to fNIRS signals obtained with normal interoptode arrangement systems, the density of measurement channels can be increased by combining it with a high-density optode arrangement technique^24^ or a high-density channel arrangement technique.^25^^,^^26^ Nonetheless, the theoretical principle of these methods involves prior knowledge of spatial or temporal differences between the cerebral functional hemodynamic response and other contaminants. If such prior knowledge were too simplified, it may cause some errors. For example, the applicability of the method based on the negative correlation between two hemoglobin species in infant brains or vascular-impaired brains is unknown.^12^ In addition, some contaminants caused by optode fluctuation often correlate with the execution of motion tasks.^7^

Taken together, these findings imply that a further method is required to allow detection of a signal exclusively arising from the cerebral tissue, with a high-density channel arrangement. In this study, we propose a new fNIRS technique for exclusively detecting cerebral signals, using neither additional optodes nor physiological prior knowledge. In the conventional fNIRS optode pair arrangement, some of the light travels into the scalp layer and exits to the air through the scalp surface. If an optical mirror is attached to the surface between the optodes in a pair, the light expected to exit will be reflected by the mirror and will again travel into the head tissue. As detailed later, this directly increases the light transiting the scalp layer and differentially changes the partial path length (PPL) of each tissue layer. By modulating the reflectance of the mirror material, such changes in PPLs cause some fNIRS signal changes. If the modulation frequency is sufficiently higher than the frequency range of the cerebral hemodynamic changes, optical characteristics in each tissue layer are likely to be constant. Thus, the difference in the signal amplitude observed through reflectance modulation can be attributed to the changes in the PPLs. Based on linear equations of optical extinction in a multilayered tissue model with different mirror reflectance conditions, the change in optical scattering and absorption in each layer can be calculated.

In the following sections, we detail the theoretical principle and experimental evaluation of this technique. In the theoretical section, we formulate the relationship between the absorbance changes in two reflectance-modulated conditions and the absorption changes in the scalp and brain layers. Then, we verify the ill-posedness in terms of the existence, uniqueness, and stability of the solution in the inverse problem for estimating the absorption changes from the absorbance changes. Experiments using a mechanically rotating mirror and a dynamic optical phantom indicated that the technique worked adequately for removing signal changes in the superficial layer and detecting signal changes in the deep layer. In the future, the reflection modulator of the rotating mirror can be replaced with a nonmechanical and flexible modulator, which can form part of the optode holder. In this way, exclusive cerebral signal measurement will be achieved, without requirements for any additional optodes, and a high-density channel arrangement will be possible by combining it with the use of an adequate modulator.

## 2 Theory

### 2.1 Feasibility Assessment through a Simulation Calculation

**Fig. 1:**
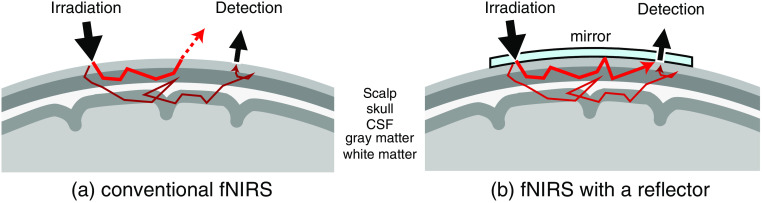
Change in light propagation in the head tissue induced by a change in scalp surface reflectance. (a) Light that reaches the scalp surface (thick red arrow) exits from the tissue and is never detected in conventional fNIRS. (b) When a mirror is placed on the scalp, the light returns to the tissue.

We propose a technique for removing scalp blood flow changes from the fNIRS signal of a single optode pair, which utilizes modulation of the scalp surface reflectance and induces changes in light propagation in tissue. Near-infrared light that enters at the scalp is propagated to be absorbed and scattered by biological tissues. In the conventional fNIRS, some of the incident light that reached the scalp surface before arriving at the detector exits to the air and is never observed by the detector [Fig. 1(a)]. On the other hand, if the scalp surface is covered with a mirror, the reflected lights again propagate in the head tissue, and some of it should be observed by the detector [Fig. 1(b)]. This means that modulation of the scalp surface reflectance can change the optical path in the head tissue. Therefore, we hypothesized that the absorbance of the head tissue is expected to be changed by the modulation of the scalp surface reflectance.

We assessed this hypothesis using a computer simulation of light propagation in the head tissue when the scalp surface reflectance, R, is 0 or 1. In the succeeding sections, the simulations with or without a surface mirror were represented as those at R=1 and R=0, respectively. The optical heterogeneity of the adult head was modeled by five-layered slabs mimicking the scalp, skull, CSF, gray matter, and white matter. The absorption coefficient and the transport scattering coefficient of a wavelength of 800 nm were assigned to each layer.^27^^–^^30^ The refractive indices of the skull, CSF, and other tissues were 1.56,^31^ 1.35,^32^ and 1.40, respectively. The optical properties and thickness of each layer were shown in Table 1. The lateral size of the model was $80\times 80\;{\mathrm{mm}}^{2}$. To our knowledge, most of the simulations on light propagation adopted homogeneous refractive index condition for the head tissue. For convenience and to allow the researchers to compare the difference in results between two refraction conditions, the same calculation was made for the optical model with the homogeneous refractive index of 1.40. As the optical model at R=0, a semi-infinite air layer was placed on the surface of the scalp layer. As the model at R=1, a vertical inversion of the five-layered model was placed on the surface of the original model. The computation of light propagation in each model was based on the diffusion equation, which was solved by the finite element method.^33^ The models were divided into homologous regular tetrahedral elements. Each vertex of the elements was used as the calculation node, and the node interval was 1 mm. The models at R=0 (R=1) included 177,147 (347,733) of nodes and 832,000 (1,664,000) of elements. The TOAST software (Department of Computer Science and the Centre for Medical Image Computing, University College London, London, United Kingdom) was customized and used for the simulation calculation. This customization of the software was detailed in literatures.^33^^,^^34^ For a case where the interval of the irradiation and the detection point is 30 mm, the detected intensity, PPL, and photon measurement density function (PMDF) were calculated.^35^ The PPL of each layer i, $l^{i}$, was calculated from the change in detected intensities while increasing the absorption coefficient of each layer, $\mu_{a}^{i}$, by 1% using

$$l^{i}=\frac{\ln(\frac{I_{\mathrm{pert},i}}{I_{\text{base}}})}{0.01\mu_{a}^{i}},$$ (1)

where $I_{\text{base}}$ and $I_{\text{pert}}$ denote the detected intensities that the baseline absorption coefficient was set and that the absorption coefficient of layer i was perturbed.

**Table 1:** Optical properties (wavelength of 800 nm) and thickness of the five-layered slabs model.

	Thickness (mm)	$\mu_{a}$ (${\mathrm{mm}}^{-1}$)	$\mu_{s}^{\prime }$ (${\mathrm{mm}}^{-1}$)	n
Scalp	3	0.018	1.94	1.40
Skull	7	0.016	1.56	1.56
CSF	2	0.004	0.23	1.35
Gray matter	4	0.036	2.23	1.40
White matter	10	0.014	9.10	1.40

Note: $\mu_{a}$, absorption coefficient; $\mu_{s}^{\prime }$, transport scattering coefficient; n, refractive index.

**Fig. 2:**
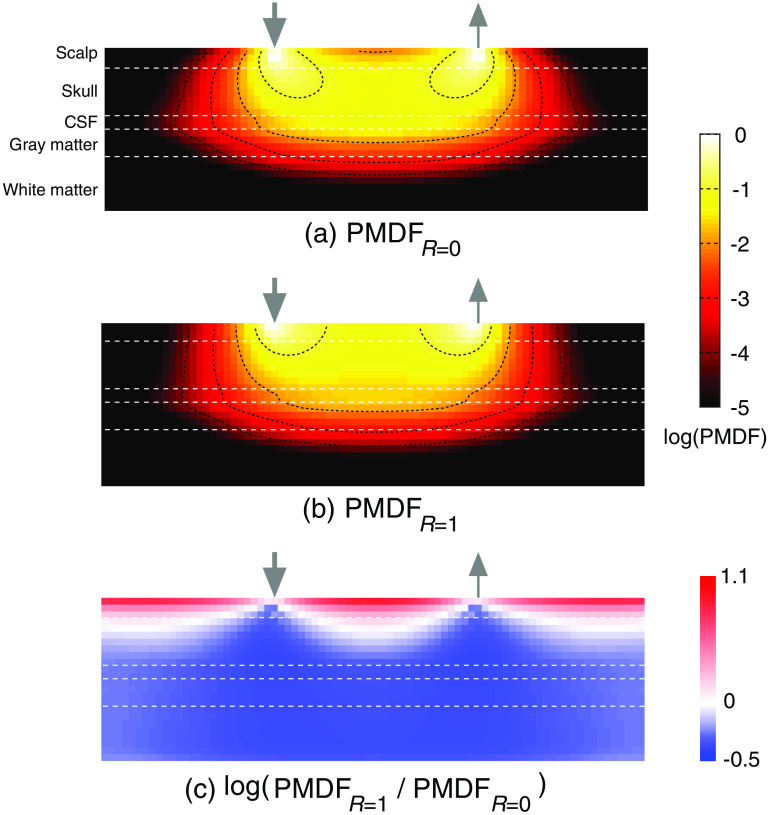
Sectional views in the distribution of the PMDF when the scalp surface reflectance, R, is (a) 0 and (b) 1, and (c) their logarithmic ratio in each voxel. The PMDF was normalized to be 1 at the point indicating their maximum value in the section. The irradiation and detection points are both perpendicular to the surface of the layered slab model and are shown with the down and up arrows, respectively. The distance between the irradiation and detection points is 30 mm. The horizontal white lines indicate the boundary between tissues.

**Fig. 3:**
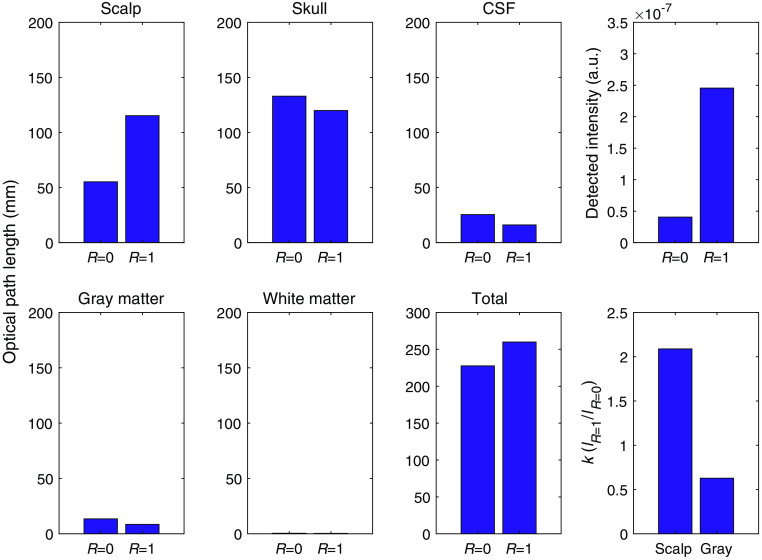
The difference in the optical path lengths in each tissue layer and the total tissue when the scalp surface reflectance, R, is 0 or 1 (left three columns). The detected intensities with and without mirror reflection are shown in the rightmost upper frame. The ratio of PPLs in the two reflectance conditions, $k=l_{R=1}/l_{R=0}$ for the scalp and gray matter is shown in the rightmost lower frame.

The simulation results (Figs. 2 and 3) showed that the light propagation in the tissue differed due to the difference in the scalp surface reflectance. The PMDF without mirror reflection (${\mathrm{PMDF}}_{R=0}$) showed a local minimum at the midpoint between the irradiation and detection points on the scalp surface [Fig. 2(a)]. On the other hand, the PMDF with mirror reflection (${\mathrm{PMDF}}_{R=1}$) showed nearly constant values in the area between the irradiation and detection points in the scalp layer [Fig. 2(b)]. The spatial distribution of the PMDF ratio, ${\mathrm{PMDF}}_{R=1}/{\mathrm{PMDF}}_{R=0}$, is shown in Fig. 2(c). The logarithmic ratio was greater than 0 mainly in the area near the surface of the scalp layer; in other areas, the logarithmic ratio was less than 0. The PPL of the scalp layer with mirror reflection was greater than that without mirror reflection; however, the PPL of the other layers was smaller in the presence of mirror reflection (Fig. 3). The detected light intensity with mirror reflection was 8.91 times greater than that without mirror reflection.

The results in the homogeneous refractive index condition were presented in Figs. S1 and S2 in the Supplemental Material. The refractive index inhomogeneity in the optical models has little effect on PPL and PMDF. Most remarkably, the PPL in the scalp layer became slightly lower in the heterogeneous refractive indices condition than the original homogeneous refractive index condition.

### 2.2 Ill-Posedness of the Proposed Method as an Inverse Problem

According to the computational assessment described in the previous section, the PPL of each tissue layer varies, due to changes in the scalp surface reflectance. It indicates that the absorbance should change with modulation of the scalp surface reflectance within the time period when the absorption change in the tissue could be assumed to be constant. The relationship between the temporal change in absorption, $\Delta \mu_{a}$, and the temporal change in absorbance at the detector, ΔA, which is called the modified Lambert–Beer law, is described by the following linear equation:

$$\Delta A_{\lambda }=\underset{i}{\sum }l_{\lambda }^{i}\Delta \mu_{a,\lambda }^{i},$$ (2)

where the suffix λ denotes the measurement wavelength, the superscript i denotes the tissue layer, and $l_{\lambda }^{i}$ denotes the PPL in the layer i at wavelength λ. Assuming that a temporal change in absorption other than those at the scalp layer (sc) and the gray matter layer (gm) is sufficiently small, the temporal change in absorbance is expressed as

$$\Delta A_{\lambda }=l_{\lambda }^{\mathrm{sc}}\Delta \mu_{a,\lambda }^{\mathrm{sc}}+l_{\lambda }^{\mathrm{gm}}\Delta \mu_{a,\lambda }^{\mathrm{gm}}.$$ (3)

Equation (3) in both cases of a high and low scalp surface reflectances are described in the following matrix equation:

$$y=\mathrm{Mx},y=[\begin{array}{c} {\Delta A}_{\text{high},\lambda }\\ {\Delta A}_{\text{low},\lambda } \end{array}],\;M=[\begin{array}{cc} l_{\text{high},\lambda }^{\mathrm{sc}} & l_{\text{high},\lambda }^{\mathrm{gm}}\\ l_{\text{low},\lambda }^{\mathrm{sc}} & l_{\text{low},\lambda }^{\mathrm{gm}} \end{array}],\;x=[\begin{array}{c} {\Delta \mu }_{a,\lambda }^{\mathrm{sc}}\\ {\Delta \mu }_{a,\lambda }^{\mathrm{gm}} \end{array}].$$ (4)

The determinant of M is

$$|M|=l_{\text{high},\lambda }^{\mathrm{sc}}\cdot l_{\text{low},\lambda }^{\mathrm{gm}}-l_{\text{high},\lambda }^{\mathrm{gm}}\cdot l_{\text{low},\lambda }^{\mathrm{sc}}.$$ (5)

From the simulation results (Fig. 3), $l_{\text{high},\lambda }^{\mathrm{sc}}>l_{\text{low},\lambda }^{\mathrm{sc}}$ and $l_{\text{low},\lambda }^{\mathrm{gm}}>l_{\text{high},\lambda }^{\mathrm{gm}}$ are expected. Thus, Eq. (5) can be transformed as follows, by introducing $\alpha =l_{\text{high},\lambda }^{\mathrm{sc}}/l_{\text{low},\lambda }^{\mathrm{sc}}$ and $\beta =l_{\text{high},\lambda }^{\mathrm{gm}}/l_{\text{low},\lambda }^{\mathrm{gm}}$:

$$|M|=l_{\text{low},\lambda }^{\mathrm{sc}}\cdot l_{\text{low},\lambda }^{\mathrm{gm}}(\alpha -\beta ),$$ (6)

where 0<β<1<a. From the above, |M|>0, i.e., M has an inverse matrix. Therefore, $\Delta \mu_{a,\lambda }^{\mathrm{sc}}$ and $\Delta \mu_{a,\lambda }^{\mathrm{gm}}$ can be obtained by solving the inverse problem of Eq. (4) as follows:

$$x=M^{-1}y,$$ (7)

where $M^{-1}$ is the inverse matrix of M. Given the elements of M by preliminary simulation calculation, as discussed later, x can be calculated using Eq. (7). Even in this case, however, the solution x will temporally fluctuate, depending on the measurement error in the data y, and its magnitude will depend on the error propagation in Eq. (7). The maximum value of the error propagation is mathematically given by the condition number of the propagation matrix. The condition number of $M^{-1}$, calculated from the PPL obtained by the simulation results, is 15.1. This magnitude is only 2.2 to 3.9 times larger than that of the propagation matrix used for calculation of hemoglobin changes in the conventionally used two-wavelength fNIRS measurements. Consequently, we confirmed that Eq. (7) has a solution, and its stability is not significantly inferior to the calculations in the conventional fNIRS.

In cases without preliminary simulation calculations, the matrix M cannot be given because its elements, PPL of different tissues, are difficult to measure directly. In our previous study in which we used multidistance probe pairs, we empirically estimated a ratio of the PPLs of the scalp layer in probe conditions with different distances without giving these values directly and successfully eliminated the influence of absorption changes in the scalp layer from fNIRS data.^11^ In the same manner, we here constructed a method for eliminating the influence of absorption changes in the scalp by empirically estimating the ratio of the PPL of the scalp layer by modulating scalp surface reflectance. From Eq. (4), the absorption change in the gray matter is described as

$${\Delta \mu }_{a,\lambda }^{\mathrm{gm}}=\frac{l_{\text{high},\lambda }^{\mathrm{sc}}}{\left|M\right|}({\Delta A}_{\text{low},\lambda }-k_{\lambda }{\Delta A}_{\text{high},\lambda }),\;k_{\lambda }=l_{\text{low},\lambda }^{\mathrm{sc}}/l_{\text{high},\lambda }^{\mathrm{sc}}.$$ (8)

Since |M|>0, as mentioned above, Eq. (8) gives a finite value of $\Delta \mu_{a,\lambda }^{\mathrm{gm}}$ with an appropriate $k_{\lambda }$ that minimizes the following cost function:

$$J(k_{\lambda })=\frac{1}{N}\overset{N}{\underset{t=1}{\sum }}\|\Delta A_{low,\lambda }^{\mathrm{cont}}(t)-k_{\lambda }\Delta A_{\text{high},\lambda }^{\mathrm{cont}}(t)\|,$$ (9)

where $\Delta A_{\text{low},\lambda }^{\mathrm{cont}}(t)$ and $\Delta A_{\text{high},\lambda }^{\mathrm{cont}}(t)$ are the time series of absorbance changes during a control measurement when the scalp surface reflectance is low or high, respectively. The control measurement is designed such that a change in absorption due to brain activation did not occur in the gray matter beneath the optode pair. The existence and uniqueness of the solution of this method are obviously ensured in Eq. (8). The stability of the solution of this method depends on the regularity of M; however, it is not directly related to its condition number. Instead of evaluation using the condition number, we evaluated the stability of this method based on a simulation calculation of the statistical accuracy of actual fNIRS measurements, in which artificial fNIRS data of a signal-to-noise ratio equivalent to that of common real data were generated and used. The simulation result revealed that this method possessed sufficient statistical accuracy for fNIRS measurements within a feasible measurement time of a few minutes to 10 min (see details in Secs. 3.2 and 4.3). Taken together, we proved that this method is applicable to actual experimental conditions.

## 3 Materials and Methods

### 3.1 Verification Procedure for the Proposed Method Using a Dynamic Phantom

**Fig. 4:**
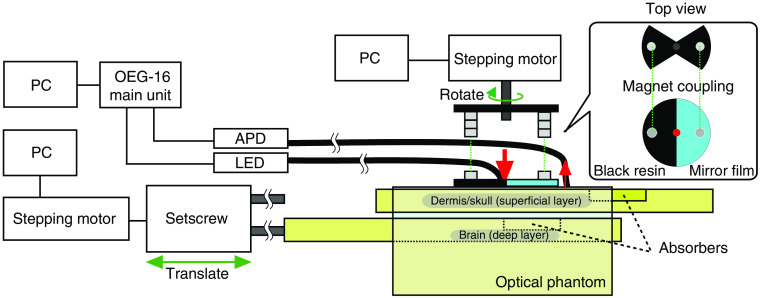
Schematic illustration of the phantom experiment. The setup consists of a reflection modulation unit, an optical measurement system, and an absorption controllable optical phantom. The reflectance on the surface of the phantom was mechanically modulated by rotating a disc that has equal parts of mirror and black body surfaces and that was magnetically coupled to a stepping motor. A pair of optical fibers connected to an LED and an APD of the fNIRS equipment (OEG-16) was attached to the surface of the optical phantom at the center and at the periphery of the reflectance modulation disc. The absorption in each superficial and deeper layer of the phantom was controlled by sliding the bar embedded in an absorber piece. The reflectance modulation and the absorbers’ motion were controlled by the programming stepping motors.

The proposed method for extracting the signal from deep layers was verified by a phantom experiment. A schematic illustration of the phantom experiment is shown in Fig. 4. A pair of irradiation and detection optodes was attached to the surface of the phantom. Optodes made of optical fibers were connected to the fNIRS equipment (OEG-16 APD, Spectratech Inc., Yokohama, Japan) of which the detector was changed to an avalanche photodiode (APD). This equipment was operationally checked and used in our previous study.^7^ The interval between the optodes was 30 mm.

We constructed a tissue-mimicking phantom in which the absorption of the superficial and deep layers could be changed independently.^36^ This phantom was comprised of four layers corresponding to the epidermis (polystyrene), dermis/skull (epoxy), CSF (acrylic), and brain (epoxy). The thicknesses of these layers were 0.3, 10, 1, and 50 mm, respectively. The optical properties of the epoxy resin were adjusted to those of the tissue in the adult head by mixing ink and titanium oxide. Each layer mimicking the dermis/skull and brain (superficial and deep layers, respectively) had a slot for laterally sliding a bar that holds an absorber piece. The absorption coefficient of the piece was higher than that of the slide bar and the layer themselves. The scattering coefficient of the slide bar was equivalent to that of the layers. One end of each slide bar was attached to a setscrew to change the position of the absorber. The motion of each bar was controlled by a stepping motor (Webmo, Cidre Interaction Design Inc., Tokyo, Japan), which rotated the setscrew.

A reflectance modulation disc was attached to the surface of the phantom. The disc-shaped reflection modulator was made of a low-reflection black poly-oxymethylene resin that functioned as an optical absorber. Half of the lower surface of the disc was covered by a mirror film (Try Tool TF1 Mirror Finish, Hasegawa Corp., Yaizu, Japan). The reflectance of the mirror film and the black resin were acquired with a spectrophotometer equipped with an integrating sphere (UV-3100; Shimadzu Corp., Kyoto, Japan) and were 0.53 and 0.08 at a wavelength of 840 nm, respectively. The disc made contact with the phantom surface and was rotated by a stepping motor (AZM46AC, Oriental Motor Co. Ltd., Tokyo, Japan) by means of a magnetic coupler. The detection and irradiation optodes were fixed to the surface of the phantom at the outer edge and the center of the disc, respectively. For the optodes, custom-made multibundled fibers of φ 2 mm made of φ 50  μm quartz were used (Moritex, corp., Saitama, Japan), and their numerical aperture was 0.56. By rotating the disc by 180 deg, the reflectance of the phantom surface at the region between the optodes was switched between high and low conditions.

**Fig. 5:**
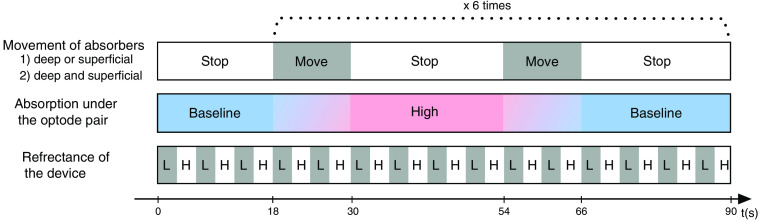
The protocol of the phantom experiment. The absorption under the optode pair was changed according to the movement of the absorbers. In the first experiment, either the deep absorber or superficial absorber was moved. In the second experiment, the deep and superficial absorbers were moved simultaneously. The surface reflectance was repeatedly switched between the high (H) and low (L) conditions by rotating the disc shown in Fig. 4.

The protocol of the phantom experiment is shown in Fig. 5. The absorption under the optode pair was changed according to the movement of the absorber. The motion sequence of the absorber was repeated six times. The reflectance was repeatedly switched between the high and low conditions by rotating the disc by 180 deg. After each rotation, the disc was stopped and remained in a stationary state for a few seconds. During the periods when both the disc and absorber were stopped, the absorbance was measured at a sampling interval of 0.65 s.

The first experiment was conducted under conditions in which the absorption in each layer was changed. Based on Eq. (9), the path length ratio, $k_{\lambda }$, was calculated from the absorbance changes when the absorption of only the superficial layer was changed. Then, another experiment was conducted in which the absorbers were simultaneously inserted into both layers. By substituting the measured absorbance change and the obtained $k_{\lambda }$ into Eq. (8), the absorption change in the deep layer, $\Delta \mu_{a,\lambda }^{\mathrm{dp}}$, was calculated from the absorbance when absorption was changed simultaneously in both the superficial and deep layers, where we set $l_{\text{high},\lambda }^{\mathrm{sc}}/|M|=1$ and $\Delta \mu_{a,\lambda }^{\mathrm{gm}}=\Delta \mu_{a,\lambda }^{\mathrm{dp}}$. To compare the proposed method with the conventional fNIRS in which surface reflectance would not exist, the absorption change obtained with the conventional fNIRS, $\Delta \mu_{a,\lambda }$, was calculated using the following equation:

$$\Delta \mu_{a,\lambda }=\frac{\Delta A_{\text{low},\lambda }}{l_{\lambda }}.$$ (10)

As a reference to evaluate the proposed method, the absorption change when the absorber was inserted only in the deep layer, $\Delta \mu_{a,\lambda }^{\mathrm{dp},\mathrm{ref}}$, was also calculated, using Eq. (10), where the reference was regarded as a true absorption change in the deep layer. The PPL in Eq. (10) was assumed to be 1, as usual, in both cases.

### 3.2 Evaluation of Applicability of the Proposed Method to the Human fNIRS Experiments

In Eq. (8), the estimated absorption changes in the gray matter layer fluctuate due to the magnification of noise by the measured absorbance change. Usually, in human experiments, such absorption changes in the gray matter layer can be assumed to fluctuate with a constant standard deviation; thus, sufficient statistical accuracy can be achieved with an appropriate sample size achieved by repetition of the trial sequence. In other words, in order for the proposed method to be used in human experiments with sufficient accuracy, trial repetition should be completed within a time achievable in human experiments. Here, we adopted sample size as a measure of the applicability of the proposed method. The minimal sample size that indicated statistical significance was evaluated through a brain activation simulation, in which an fNIRS experiment, comprised of a certain number of control and task trials, was simulated.

In this simulation, the amplitude of the absorbance change caused by brain activation was fixed to be equivalent to that of the adult motor cortex by referring to a previous fNIRS study. To determine the absorbance change, the amplitudes and standard deviations of the changes in path-length-dependent oxyhemoglobin and deoxyhemoglobin concentrations were obtained as 0.10±0.08 and −0.05±0.04 mM·mm, respectively, from a literature.^12^ The amplitude of the absorbance change in the brain activation simulation was calculated using the following equation:

$$\Delta A=\varepsilon_{\mathrm{oxy}}\Delta C_{\mathrm{oxy}}+\varepsilon_{\mathrm{deoxy}}\Delta C_{\mathrm{deoxy}},$$ (11)

where $\varepsilon_{\mathrm{oxy}}$ and $\varepsilon_{\mathrm{deoxy}}$ were molar extinction coefficients of oxyhemoglobin and deoxyhemoglobin, respectively, and $\Delta C_{\mathrm{oxy}}$ and $\Delta C_{\mathrm{deoxy}}$ were amplitude of path-length-dependent oxyhemoglobin and deoxyhemoglobin concentrations, respectively. The molar extinction coefficients at a wavelength of 800 nm were chosen from the literature.^37^ This calculated amplitude of absorbance change was regarded as that in Eq. (2). The values without scalp surface reflection simulated in Sec. 2 were used as the PPL in the scalp and gray matter layers. The mean amplitudes of the absorption changes in each layer, $\overset{¯}{\Delta \mu_{a,\lambda }^{l}}$, were given so that $l_{\lambda }^{i}\overset{¯}{\Delta \mu_{a,\lambda }^{l}}$ were equivalent to each other. The sampling interval of the absorbance change was set as 0.1 s. The absorbance changes in the control period (involving no brain activation) of 10 s and in the task period (involving brain activation) of 10 s were generated, to which noises with a standard deviation calculated from the standard deviation of the change in hemoglobin concentration described above was added. The contrast-to-noise ratio of the absorbance changes (amplitude divided by standard deviation) was 0.59. The absorbance change with scalp surface reflection was also calculated in the same manner, where the values obtained in the simulation with the scalp surface reflection were used as the PPL. The same number of samples in the control and task periods was used for calculating t-values in unpaired t-test comparisons of the two groups.

## 4 Results

### 4.1 Changes in PPLs Induced by Surface Reflectance Changes

**Fig. 6:**
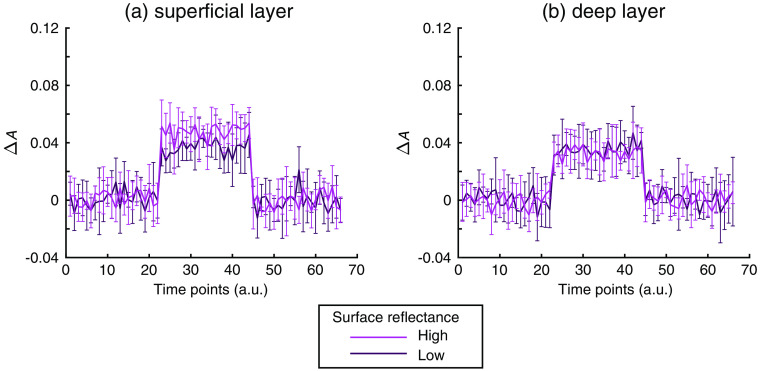
Absorbance changes (ΔA) induced by individual insertion of the absorber in the (a) superficial layer and (b) the deeper layer. The light and dark lines in each frame indicate the change in the conditions of high and low surface reflectances, respectively. Error bars indicate standard deviations of ΔA over six trials.

The absorbance changes when each absorber was inserted separately in the superficial and the deep layers are shown in Fig. 6. In the former case [Fig. 6(a)], the absorbance increased more markedly when the phantom surface reflectance was higher. However, in the latter case [Fig. 6(b)], no difference in the increase depending on the phantom surface reflectance was observed. Since the absorption change in each layer was constant between the two measurement conditions with different phantom surface reflectance values in each case, the absorbance change was attributed to the change in the PPL of each layer under Eq. (3). Therefore, the results demonstrated that the PPL of the superficial layer was changed more markedly by modulation of the surface reflectance than was that of the deep layer.

### 4.2 Removal of Influence by Superficial Absorption Change Using Surface Reflectance Modulation

**Fig. 7:**
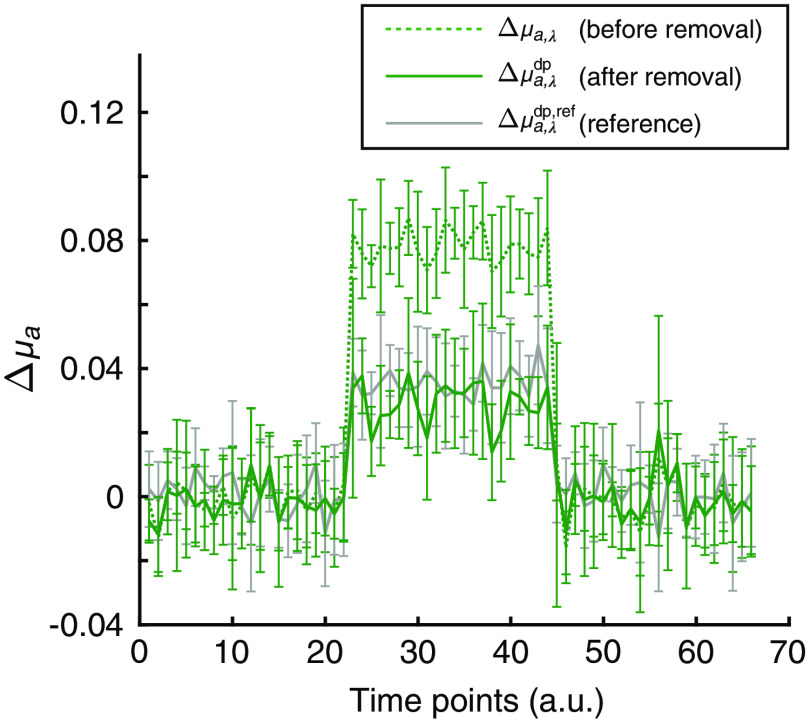
Absorption changes obtained by phantom experiments. The dotted green line indicates the absorption change $\Delta \mu_{a,\lambda }$ in the conventional method as calculated by Eq. (10), using absorbance change when the absorbers were inserted simultaneously in both the superficial and deep layers of the phantom. The solid green line indicates the absorption change $\Delta \mu_{a,\lambda }^{\mathrm{dp}}$ of the deep layer as extracted by the proposed method, i.e., Eq. (8), using the same absorbance change as used for calculating the dotted line. The reference data $\Delta \mu_{a,\lambda }^{\mathrm{dp},\mathrm{ref}}$, indicated with a solid gray line, are given by changing the absorption only in the deep layer. Error bars indicate standard deviations of the absorption change over six trials.

Absorption changes obtained by the phantom experiment are shown in Fig. 7. The absorption changes $\Delta \mu_{a,\lambda }$ and $\Delta \mu_{a,\lambda }^{\mathrm{dp}}$, before and after removal of the absorption change in the superficial layer, respectively, were calculated from the absorbance changes when the absorber was inserted into both layers simultaneously. In other words, the $\Delta \mu_{a,\lambda }$ and $\Delta \mu_{a,\lambda }^{\mathrm{dp}}$ were calculated by the conventional method [Eq. (10)] and the proposed method [Eq. (8)], respectively. The absorption change $\Delta \mu_{a,\lambda }^{\mathrm{dp},\mathrm{ref}}$ when the absorber was inserted solely into the deep layer was also calculated by the conventional method using Eq. (10) as a reference for evaluation of the proposed method. $\Delta \mu_{a,\lambda }^{\mathrm{dp},\mathrm{ref}}$ was $0.0355\pm 0.0031\;{\mathrm{mm}}^{-1}$. Before removing the influence of the absorption change in the superficial layer, $\Delta \mu_{a,\lambda }$ was $0.0803\pm 0.0070\;{\mathrm{mm}}^{-1}$, which was significantly larger than $\Delta \mu_{a,\lambda }^{\mathrm{dp},\mathrm{ref}}$ ($p<10^{-36}$). After removing the influence of the absorption change of the superficial layer by the proposed method, in contrast, $\Delta \mu_{a,\lambda }^{\mathrm{dp}}$ was $0.0311\pm 0.0027\;{\mathrm{mm}}^{-1}$, which was not significantly different from $\Delta \mu_{a,\lambda }^{\mathrm{dp},\mathrm{ref}}$ (p>0.01). Therefore, the error from the reference data was reduced to less than 1/10th by applying the proposed method. These results strongly indicate that the influence of the absorption change in the superficial layer was successfully removed from the absorbance when the absorption was simultaneously changed in the superficial and deep layers.

### 4.3 Applicability of the Proposed Method to Human fNIRS Experiments

**Fig. 8:**
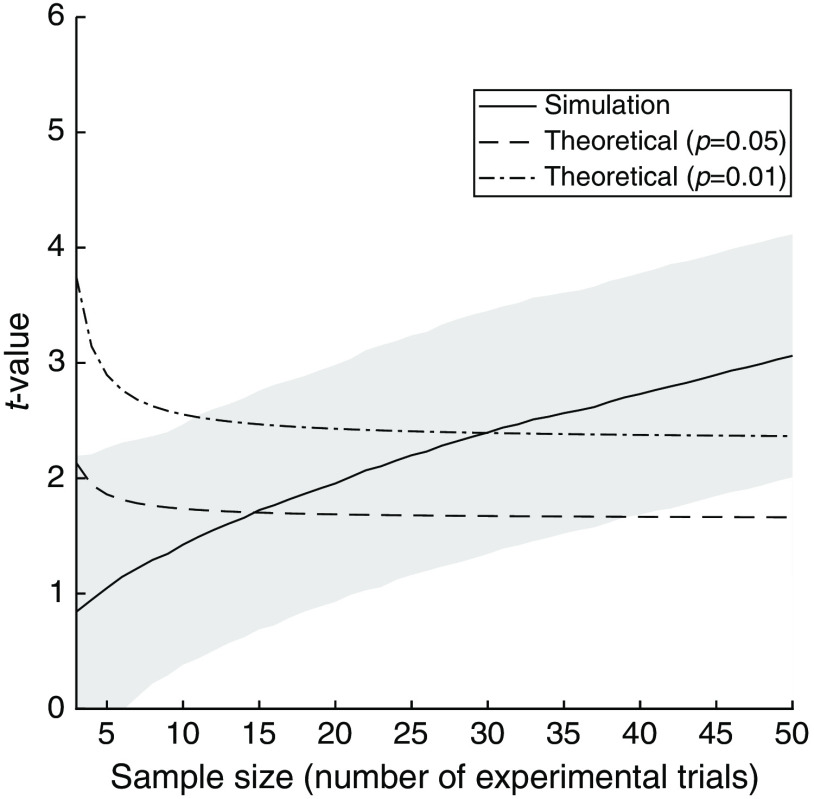
Number of experimental trials required to achieve statistical significance based on two-sample t-tests of absorption changes between control and task periods. The solid line and gray area indicate average and standard deviation over the 1000 simulation trials, respectively, where the measurement values in each trail had a normal distribution. The dashed and chain lines indicate the p=0.01- and p=0.05-equivalent t-value curves, depending on the number of experimental trials, respectively.

We conducted a brain activation simulation to calculate the minimum number of experimental trials required to achieve statistical significance in a two-sample t-test for absorption changes between control and task periods. Figure 8 shows the results. For the typical fNIRS data, which have a similar signal-to-noise ratio as in human measurements, the number of trials (sample sizes) required to achieve t-values with significance levels of p=0.05 and p=0.01 was 15 and 30, respectively. In typical human task experiments, tasks of several tens of seconds are repeated 10 to 20 times and the total experimental sequence requires several minutes to several tens of minutes. The number of trials required for statistical accuracy shown in Fig. 8 sufficiently matches this task repetition number. Therefore, the proposed method is applicable to human experiments with a standard design.

## 5 Discussion

In this study, we proposed a technique for removing scalp blood flow changes from the fNIRS signal, assuming that the PPL of each tissue layer changes by switching the reflectance of the scalp surface. Intuitively, the optical path lengths could increase when the scalp surface reflectance is high, because more photons re-enter into and travel through the head tissues to the detector. However, the simulation results in Fig. 3 showed that the PPLs other than that in the scalp layer decreased, whereas that in the scalp layer increased. When the scalp surface reflectance is high, the photons detected by the detector can be grouped into two categories: those that were reflected and those that were not reflected by the mirror. The detector receives the photons not reflected by the mirror, similarly to that in conventional fNIRS; moreover, it additionally receives the photons reflected by the mirror. The photons reflected by the mirror re-enter into the tissue at individual reflection points. For these reflected photons, the distances between their reflected points and the detector are shorter than the interval between the source and detector optodes. The propagation of such photons after their re-entry into the tissue resembles a situation in which the interval between optodes is smaller. Comparing such propagation to the no-reflection case, the ratio of the PPL to the total optical path length is larger in the superficial scalp layer, but is smaller in the deep brain layer.^13^ Consequently, this may explain why the PPLs were larger in the scalp layer and smaller in the other deeper layers than seen in the low reflectance condition. The PPL difference between the two surface reflectance conditions may be changed to an extent with the simulation condition such as optical properties and thickness of tissue layers. However, such tendencies of responses of PPLs to reflectance modulation will be held in any case. These tendencies (0<β<1<α) ensure the positiveness of |M| in Eq. (6); thus, the proposed method can constantly separate the data into those originating from superficial and from deep layers. Note, however, that the phantom experiment (Fig. 6) did not indicate a distinct difference in the PPL in the deep layer with a change in surface reflectance. This may be attributed to the magnitude of the standard deviation in the measurement. Such large standard deviation of the signal relating to the weakness of detected light obviously disturbs the difference of PPL in gray matter that is observed. In addition, the monochromaticity of irradiation light and the difference in refractive indices between the layers of the phantom may influence this issue. The irradiation light was supposed to be monochromatic in our simulation study; however, the light emission diode (LED) light source used in the phantom experiment has a finite spectral dispersion. This property of irradiation source naturally increases the deviation in PPLs of the detected lights that would decrease the experimental sensitivity to the difference in PPL. Under such conditions, the refractive index heterogeneity in the phantom would synergistically enlarge more the deviation in PPLs to decrease the sensitivity to the difference in PPL. Fortunately, in actual human measurements, the refractive index heterogeneity in the human head tissues is supposed to be smaller than that in the present phantom. Moreover, such synergistic influences from these factors will be limited using monochromatic light source such as laser. In addition, the limited area covered by the mirror in the experiment as compared to the simulation may have caused the difference from the simulation result. The difference in reflectance between the conditions with and without mirrors in the phantom experiment was less than half of that in the simulation, which may also explain the difference between the simulation and experimental results. Nevertheless, the proposed method successfully removed the absorption changes of the superficial layer in the phantom experiments (Fig. 7).

In the proposed method for estimating absorbance changes in the gray matter layer, we need to determine the value of $k_{\lambda }=l_{\text{low},\lambda }^{\mathrm{sc}}/l_{\text{high},\lambda }^{\mathrm{sc}}$ in Eq. (8) in some empirical manner. For the determination of $k_{\lambda }$, simulation calculations may be adopted. In this approach, the structural model should be constructed using anatomical images from MRI;^38^ however, anatomical imaging may not be possible with every fNIRS experiment. Although representative values of optical properties of tissues can be obtained by referring to the literature,^39^ no method has been established to obtain accurate values for individual subjects. Instead, we have proposed another method that provides changes in the absorption of the gray matter layer based on measurement data only. We employed the method without PPL estimation assuming that $l_{\text{high},\lambda }^{\mathrm{sc}}/|M|=1$ in Eq. (8). This assumption causes the change in absorption of the scalp layer to be unknown and causes a systematic error, so-called crosstalk, in the change in absorption of the gray matter layer according to the wavelength used. On the other hand, the difference from the reference data was significantly reduced using the proposed method, as shown in the results of the phantom experiment. Therefore, the method using the path length ratio is sufficiently practical and effective in removing scalp absorption changes, even when the PPL is not provided.

The applicability of the proposed method was validated only under limited conditions in this study. The results shown may vary modestly with the structure of tissues and their optical properties, the baseline stability in absorption changes, and signal-to-noise ratios in the measurement system. However, for human fNIRS measurements under prevalent conditions, the results obtained with such measurements will not be very different from those obtained in this study. Familiar tasks in human experiments such as verbal fluency, verbal working memory, finger tapping, and visual checker-board tasks generally indicate higher contrast-to-noise ratios of the hemoglobin concentration changes than that indicated in this simulation,^8^ which suggests that the proposed method will be applicable to detect the functional activations evoked by these tasks. Thus, the proposed method seems to work with most human fNIRS measurements.

**Fig. 9:**
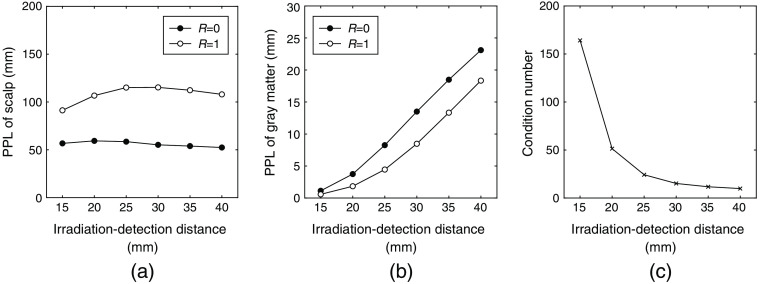
Theoretical applicability of the proposed method with various irradiation-detection distances in the adult head model. The PPLs for (a) the scalp and (b) gray matter with scalp surface reflectance R=0 and 1 were utilized to calculate (c) the condition number for the matrix M in Eq. (4). The conditions of the light propagation simulation to calculate the PPLs, other than the irradiation-detection distances, were given in Sec. 2.

Several issues need to be verified in using the proposed method for human fNIRS measurements. The most important issue may be the optimum irradiation-detection distance for the proposed method. Figure 9 presented the theoretical applicability of the proposed method with various irradiation-detection distances through simulation of the light propagation in the adult head model. The condition number in Fig. 9 decreased with increasing irradiation-detection distance. A very short distance between irradiation and detection optodes such as 15 mm increases the condition number, i.e., the ill-posedness of the proposed method to magnify the noise propagation in Eq. (7). In the practical measurement, in contrast, the SNR of the detected light will be decreased with increasing the irradiation-detection distance. Both increase in the required trial number for achieving the significant t-level. Consequently, the range of about 25 to 40 mm will be adoptable for the irradiation-detection distance in the measurement using the proposed method for adult human experiments. The mechanical rotating mirror used in the phantom experiment is less practical for fixing to a human head. In its place, a reflectance modulation device for human fNIRS measurements can be made of soft materials that can electrically modulate reflectance, such as a switchable mirror on a flexible sheet.^40^ In addition, the strong absorption of the hair between the device and the scalp may prevent the utility of the proposed method. In particular, the utility of the proposed method is completely lost if the light arriving at the scalp surface completely disappears due to absorption by the hair. Preparation such as partitioning hair and exposing the scalp directly under the device is needed to use the proposed method effectively in regions with dense hair. Shaving the subject’s head could be one solution to be able to apply the proposed method; however, the burden on the subject might be excessive. Partitioning the hair and partially exposing the scalp surface may be effective in the practical application of the proposed method, although its effectiveness may be limited. Further studies are needed to reveal the extent of the effectiveness of the partial exposure of the scalp.

The proposed method is extremely effective for removing scalp blood flow changes in areas where light is not obstructed by hair, such as the human forehead and neonatal head. In particular, for the neonatal head, the irradiation and detection optodes should be attached more densely, with a shorter interval, than for the adult head, because the brains of these subjects are smaller than those of adults. In such densely packed conditions, the multidistance optode arrangement to remove the scalp blood flow effect may not be executed. It is unknown whether removal of scalp blood flow changes based on physiological prior knowledge can appropriately work for fNIRS data of newborns and infants, as recent studies have suggested that neurovascular coupling in neonates and preterm born infants is different from that in adults.^41^ Because the proposed method requires neither additional optodes nor any physiological prior knowledge in cerebral hemodynamics, it is the most suitable technique for removing the scalp blood flow effect in fNIRS data of neonates and infants.

## 6 Conclusion

We proposed an fNIRS technique that exclusively detects cerebral functional hemodynamic changes using reflectance modulation of the scalp surface. The theoretical feasibility of the proposed method was proven by a simulation calculation of light propagation. A phantom experiment demonstrated that the influence of the absorption change in the superficial layer was successfully reduced by the proposed method using only measurement data. A brain activation simulation study demonstrated that the proposed method was applicable to human experiments with standard designs in terms of achieving statistical significance in an acceptable experimental time-frame. The removal of the scalp blood flow effect by the proposed technique will increase the quality of fNIRS data, particularly in the assessment of neonates and infants, which requires a dense optode arrangement.
