# Supplementary material for: Exclusive detection of cerebral hemodynamics in functional near-infrared spectroscopy by reflectance modulation of the scalp surface
Source: J Biomed Opt. 2020 Aug 5;25(8):087001. doi: 10.1117/1.JBO.25.8.087001 (PMC7403450; doi:10.1117/1.JBO.25.8.087001)
Supplement: Supplementary file 1 [file JBO_025_087001_SD001.pdf]

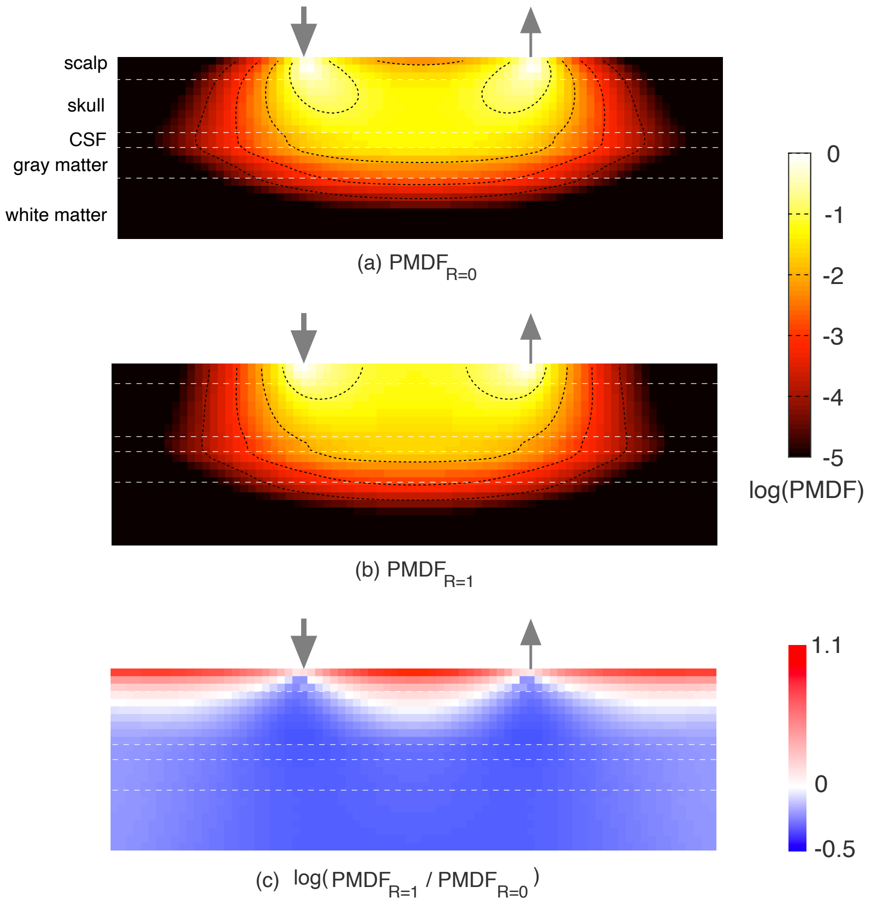

Fig. S1 Sectional views in the distribution of the photon migration density function (PMDF) when the scalp surface reflectance, R, is 0 (a) and 1 (b), and their logarithmic ratio in each voxel (c). The irradiation and detection points are both perpendicular to the surface of the layered slab model and are shown with the down and up arrows, respectively. The distance between the irradiation and detection points is 30 mm. The horizontal white lines indicate the boundary between tissues. The PMDF was calculated on the optical model with the homogeneous refractive index of 1.40. In the simulation for obtaining the results shown in this figure, conditions and parameters other than the refractive index were exactly the same as those in the simulation for obtaining the results shown in Fig. 2.

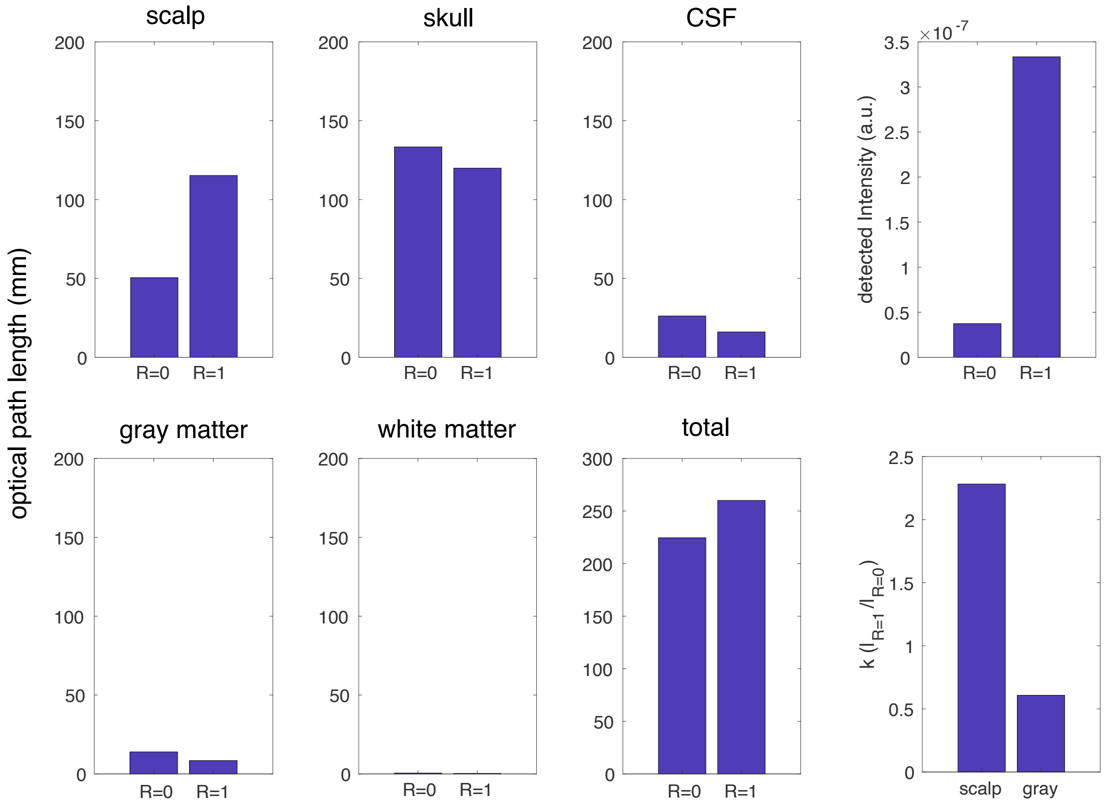

Fig. S2 The difference in the optical path lengths in each tissue layer and the total tissue when the scalp surface reflectance, R, is 0 or 1 (left three columns). The detected intensities with and without mirror reflection are shown in the rightmost upper frame. The ratio of partial path lengths in the two reflectance conditions, $k = l_{R=1}/l_{R=0}$ for the scalp and gray matter is shown in the rightmost lower frame. These results were calculated on the optical model with the homogeneous refractive index of 1.40. In the simulation for obtaining the results shown in this figure, conditions and parameters other than the refractive index were exactly the same as those in the simulation for obtaining the results shown in Fig. 3.
